# Effectiveness of distal tibial osteotomy with distraction arthroplasty in varus ankle osteoarthritis

**DOI:** 10.1186/s12891-020-3061-7

**Published:** 2020-01-14

**Authors:** Koji Nozaka, Naohisa Miyakoshi, Takeshi Kashiwagura, Yuji Kasukawa, Hidetomo Saito, Hiroaki Kijima, Shuichi Chida, Hiroyuki Tsuchie, Yoichi Shimada

**Affiliations:** Department of Orthopedic Surgery, Akita University Graduate School of Medicine, Hondo, Akita 010-8543 Japan

**Keywords:** Distal tibial osteotomy, Medial ankle arthritis, Joint distraction, Circular external fixator

## Abstract

**Background:**

In highly active older individuals, end-stage ankle osteoarthritis has traditionally been treated using tibiotalar arthrodesis, which provides considerable pain relief. However, there is a loss of ankle joint movement and a risk of future arthrosis in the adjacent joints. Distraction arthroplasty is a simple method that allows joint cartilage repair; however, the results are currently mixed, with some reports showing improved pain scores and others showing no improvement. Distal tibial osteotomy (DTO) without fibular osteotomy is a type of joint preservation surgery that has garnered attention in recent years. However, to our knowledge, there are no reports on DTO with joint distraction using a circular external fixator. Therefore, the purpose of this study was to examine the effect of DTO with joint distraction using a circular external fixator for treating ankle osteoarthritis.

**Methods:**

A total of 21 patients with medial ankle arthritis were examined. Arthroscopic synovectomy and a microfracture procedure were performed, followed by angled osteotomy and correction of the distal tibia; the ankle joint was then stabilized after its condition improved. An external fixator was used in all patients, and joint distraction of approximately 5.8 mm was performed. All patients were allowed full weight-bearing walking immediately after surgery.

**Results:**

The anteroposterior and lateral mortise angle during weight-bearing, talar tilt angle, and anterior translation of the talus on ankle stress radiography were improved significantly (*P* < 0.05). Signal changes on magnetic resonance imaging also improved in all patients. Visual analog scale and American Orthopedic Foot & Ankle Society scores improved significantly (*P* < 0.05), and no severe complications were observed.

**Conclusion:**

DTO with joint distraction may be useful as a joint-preserving surgery for medial ankle osteoarthritis in older patients with high levels of physical activity.

**Level of evidence:**

Level IV, retrospective case series.

## Background

To date, ankle arthrodesis or total ankle arthroplasty has been performed in patients with progressive or end-stage ankle osteoarthritis. With the emergence of a super-aged society and an increased number of older patients with high levels of physical activity, joint-preserving surgery has become has become increasingly popular. The surgical treatment of ankle osteoarthritis varies according to the stage. In patients with stage II and IIIA ankle osteoarthritis, low tibial osteotomy (LTO) is indicated for correcting the alignment of the lower end of the tibia surface [1–5], whereas total ankle arthroplasty or arthrodesis is generally indicated for patients with end-stage arthropathy (stages IIIB and IV) [6–9]. Intraarticular deformities may also be treated with total ankle arthroplasty, and distraction arthroplasty is indicated for patients with stage III or IV arthropathy [10–14]. In younger-aged patients with post-traumatic osteoarthritis, distraction arthroplasty is often performed with an external fixator. However, Tellisi and Fragomen [15] reported that in terms of joint preservation in the osteoarthritic ankle, older patients (more than 60 years old) tend to have better outcomes with distraction arthroplasty than their younger counterparts. Horn and Fragomen [16] also reported that supramalleolar osteotomy using circular external fixation is an effective method for correcting distal tibial deformities in the adult population. Plafond-plasty is also well indicated for various stages of intra-articular varus ankle osteoarthritis (including stage IIIB) associated with ankle instability [17].

Distal tibial osteotomy (DTO) is a type of joint-preserving surgery allowing patients to reacquire ankle stability and achieve weight-bearing; hence, it has been reported that DTO using a site with remaining healthy cartilage is indicated for stage II-IIIB arthropathy. However, to our knowledge, there are no reports on DTO with joint distraction using a circular external fixator. Therefore, this study aimed to examine the effect of DTO on ankle osteoarthritis with joint distraction using a circular external fixator.

## Methods

Twenty-one patients (7 males and 14 females; mean age: 68.2 years; age range: 60–80 years) with medial ankle osteoarthritis (Takakura classification stage IIIA: 4 cases and stage IIIB: 17 cases), who underwent DTO with joint distraction using a circular external fixator and had undergone ≥2 years of follow-up, were included in the study. Overall, 17, 2, 1, and 1 patients had primary ankle osteoarthritis, ankle rheumatoid arthritis, post-traumatic ankle osteoarthritis (tibial shaft fracture), and polio-related ankle osteoarthritis, respectively. The left side was affected in 12 patients. The patients were all highly active people, and included farmers, manual laborer, and sports enthusiasts; they had chronic ankle pain with swelling, stiffness, and difficulty in walking, all of which are symptoms of ankle osteoarthritis.

The mean period to bone union was 85.0 days (range, 77–121 days). The mean period with an external fixator was 89.2 days (range, 80–128 days) and the mean follow-up period was 3.2 years (range, 2–9 years). Magnetic resonance imaging (MRI) was performed at a mean of 11.2 months (range, 10–14 months) after frame removal. Pre- and postoperative images were obtained, as well as the latest X-ray. The Patient Archiving and Communication System (PACS) software was used, which allowed for remarkably consistent measurements on radiographs. We confirmed the absence of errors on the AP, lateral, and mortise views of the radiographs on every occasion, using the scale in PACS. We also checked for weightbearing on AP, lateral, and mortise views of the radiographs every 1 to 3 months, and assessed the arthropathic changes such as the appearance of loss of joint space, subchondral sclerosis, cysts, and eburnation.

### Surgical methods

**Fig. 1:**
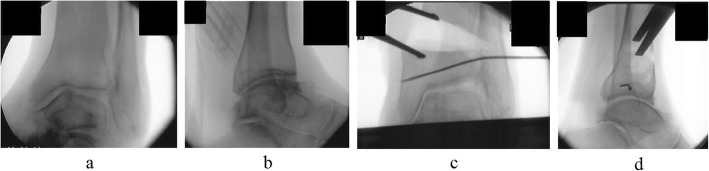
Osteotomy fluoroscopic images. **a** Varus tilt of the talus on anteroposterior fluoroscopic imaging before osteotomy. **b** Lateral fluoroscopic image before osteotomy. **c** Fluoroscopic image after osteotomy. The deformity has been corrected in the coronal plane. **d** Fluoroscopic image taken after osteotomy. Correction is performed until the radiographic signs of subluxation disappear on lateral fluoroscopy imaging

**Fig. 2:**
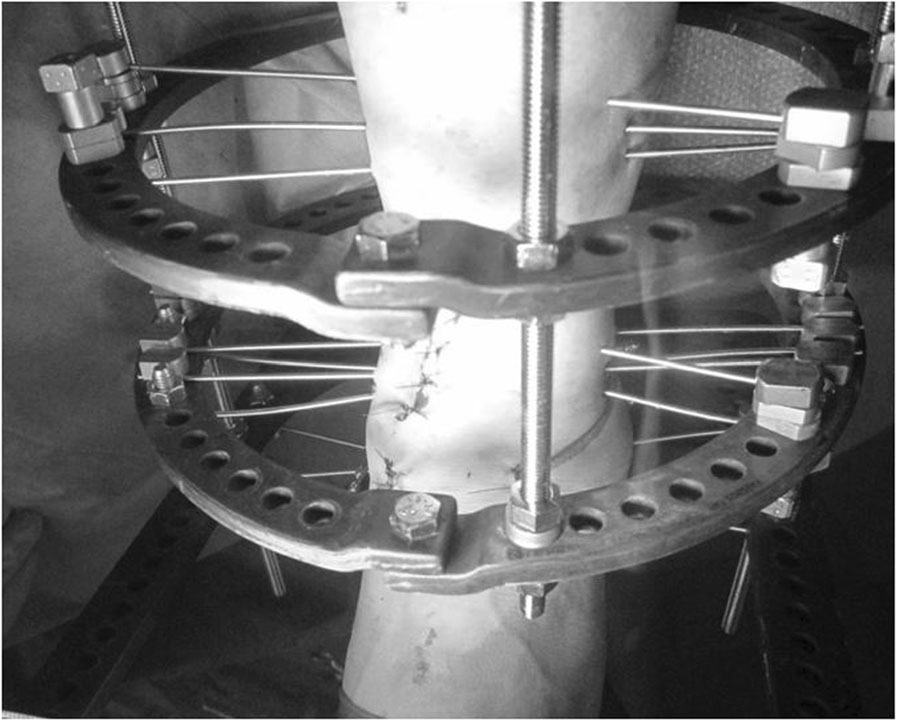
The distal bone fragment is fixed in place just above the ankle with six straight wires

**Fig. 3:**
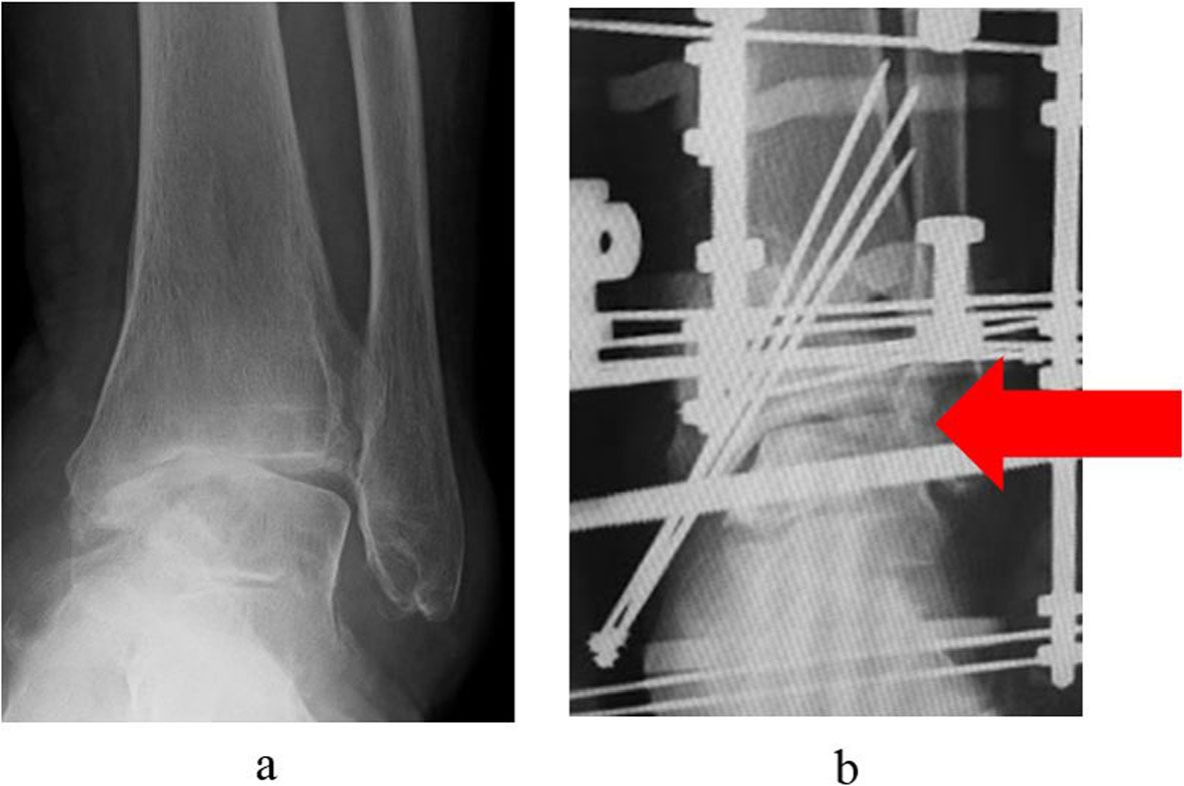
A foot ring was used for joint distraction. **a** Before joint distraction. **b** After joint distraction (arrows)

**Fig. 4:**
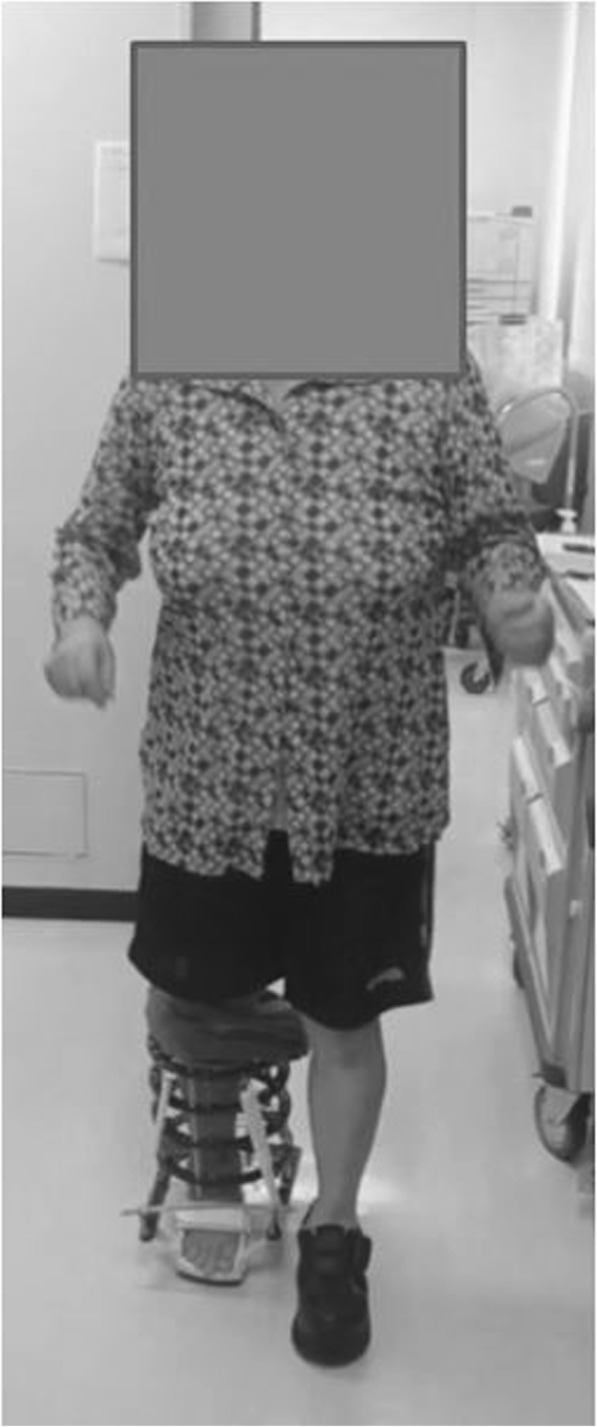
Full weight-bearing walking immediately after surgery

**Fig. 5:**
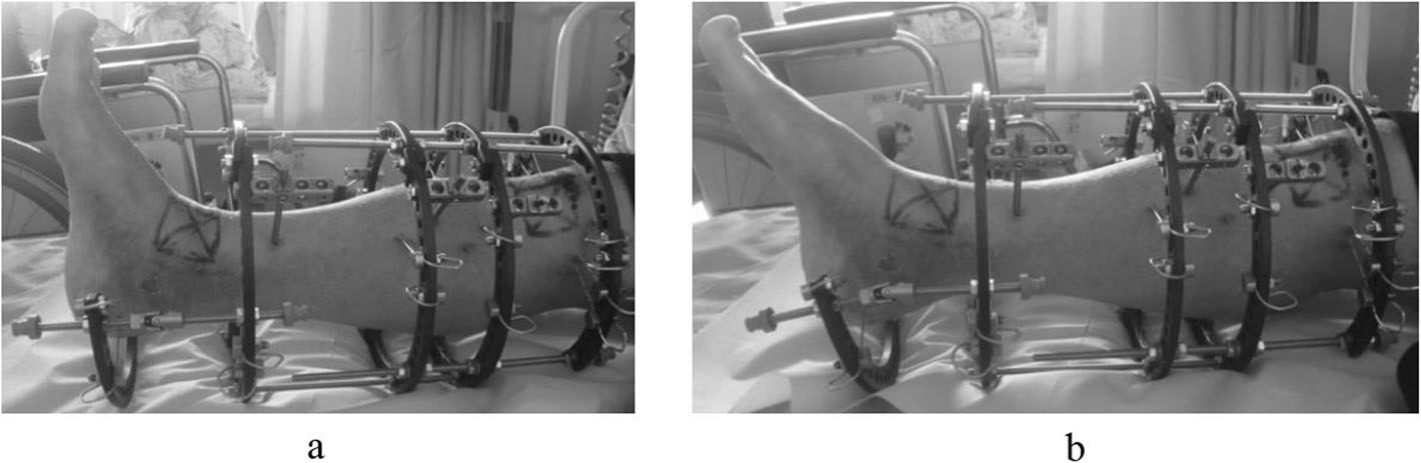
Patients treated with joint distraction perform articulation while wearing an external fixator, allowing for an increased range of motion (ROM). **a** Dorsiflexion. **b** Plantarflexion

An external fixator was used in all patients. Ankle arthroscopy was first performed, following which a microfracture procedure was performed under arthroscopy after synovectomy. Osteotomy was then performed to create an opening from the medial aspect of the distal tibia, towards the tibiofibular joint. The osteotomy line was defined from a point at the medial cortex, 3 cm proximal to the joint line, to a point on the lateral tibial cortex 1 cm proximal to the joint line, and an opening was created in the tibia. The deformity was corrected in the coronal plane after the surgeon pushed on the ankle until it was stable or until the talus was perpendicular to the tibia. Further correction was performed until radiographic signs of subluxation disappeared on the lateral fluoroscopy image (Fig. 1). An ipsilateral iliac bone was subsequently transplanted into the opening. In patients who continued to experience less than 10 degrees of ankle dorsiflexion after opening-wedge correction osteotomy, Vulpius-type Achilles tendon lengthening techniques were additionally required. In addition to one foot ring, four circular external rings were applied from the proximal tibia to immediately above the ankle. The distal bone fragment was fixed in place immediately above the ankle using six straight wires (Fig. 2). During fixation with a circular external fixator, foot ring fixation (for the calcaneus) and joint distraction of approximately 5.8 mm were also performed (Fig. 3a, b) [13]. We also reduced the width of distraction during surgery when a large opening (i.e., approximately 20 mm) was required at the osteotomy site, and the tension of the soft tissue was strong. To avoid tibial nerve palsy, we gradually applied slight distraction to the ankle while checking the condition of the soft tissue and the ability to move the ankle and toes after surgery; this was continued until a 5.8-mm distraction was achieved in the ankle joint. If the radiographic joint space during distraction arthroplasty showed a minimum of 5.8 mm distraction gap, it ensured no contact between the joint surfaces during full weight-bearing. All patients were allowed full weight-bearing walking immediately after surgery (Fig. 4). Patients treated with joint distraction performed articulation while wearing an external fixator, which reportedly increases the range of motion (ROM), promotes regeneration of a good articular surface, and facilitates maturation and regeneration of fibrocartilage. Hinges were placed along the axis of ankle motion, which was evaluated using anteroposterior (AP) and lateral fluoroscopy images to ensure proper placement. Two universal hinges were attached on either side of the ring using threaded rods (Fig. 5).

### Evaluation

**Fig. 6:**
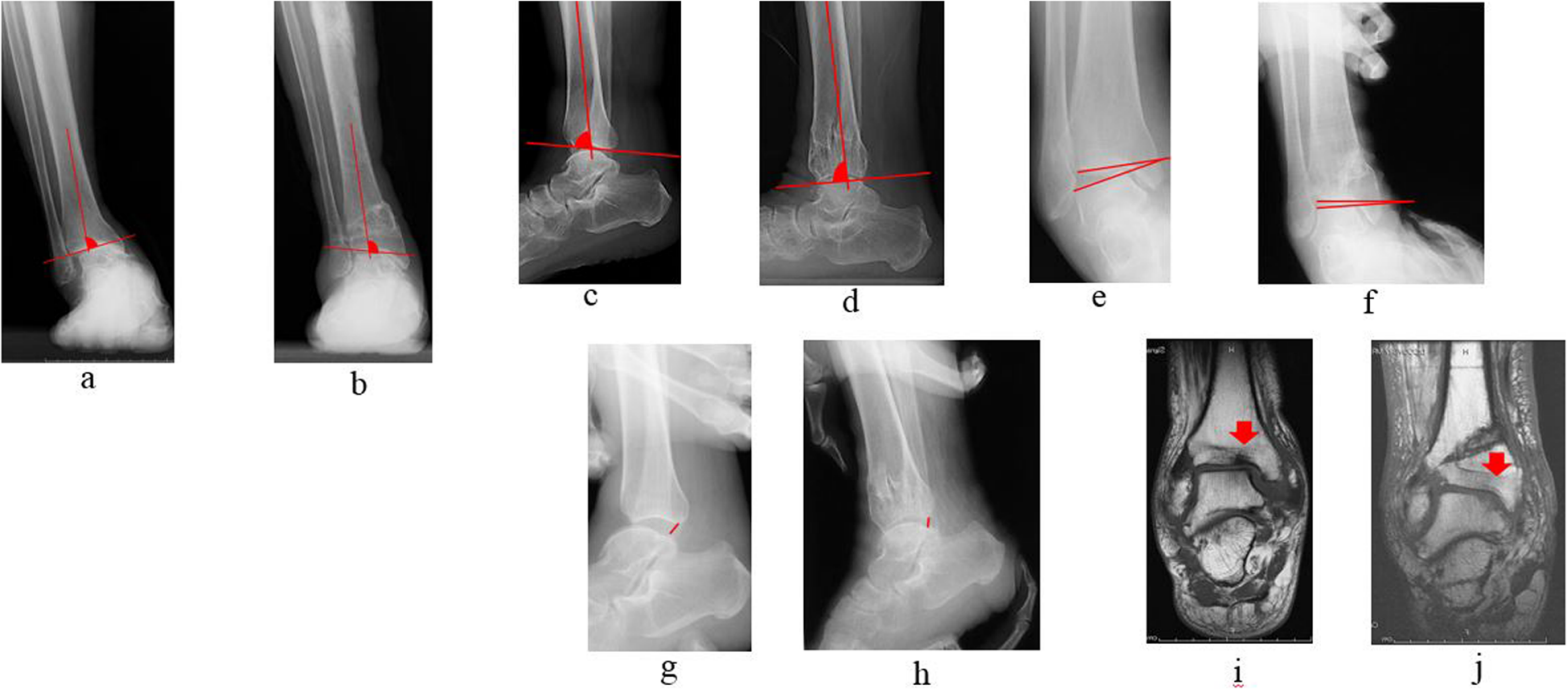
Pre- and postoperative images. **a** Preoperative antero-posterior mortise angle on X-ray. **b** Postoperative antero-posterior mortise angle on X-ray. **c** Preoperative lateral mortise angle on X-ray. **d** Postoperative lateral mortise angle on X-ray. **e** Preoperative talar tilt angle on ankle stress radiography. **f** Postoperative talar tilt angle on ankle stress radiography. **g** Postoperative anterior translation of the talus on ankle stress radiography. **h** Postoperative anterior translation of the talus on ankle stress radiography. **i** Signal changes (arrow) observed on magnetic resonance imaging (MRI) before osteotomy. **j** Signal changes (arrow) on MRI disappeared after osteotomy

Static parameters were assessed using pre- and postoperative radiographic images including the AP and mediolateral (ML) mortise angles during weight-bearing (Fig. 6). Dynamic parameters were also assessed using pre- and postoperative radiographic measurements of manual mortise ankle stress, which were available for all patients preoperatively. Both lateral and mortise stress radiographs were obtained at the final follow-up visit. The tibiotalar tilt was measured on the mortise stress view of the ankle. Anterior displacement of the talus with respect to the tibia was assessed on the lateral stress radiograph. Varus instability was assessed as the degree of talar tilt and measured as the angle (in degrees) between the superior surface of the talus and the tibial plafond on a mortise radiograph of the ankle. Maximal manual pressure was used to exert an inversion/varus force across the ankle joint. Anterior instability was assessed as the anterior translation of the talus (the distance between the posterior edge of the tibial articular surface and the posterior edge of the talar trochlea) on a lateral radiograph stress test. Radiographic anterior translation of the talus was assessed by measuring the nearest distance from the posterior edge of the distal tibial plafond to the posterior edge of the joint surface on the talar dome. Maximal manual pressure was used to exert anterior translation of the talus (Fig. 6).

The rate of improvement in MRI signal changes (preoperative versus postoperative) was also determined (Fig. 6). MRI was performed on a 1.5-T system; T1and T2-weighted sequences were utilized to assess signal changes.

The rate of adjacent joint arthritis was measured using pre- and post-operative visual analog scale (VAS) and American Orthopedic Foot and Ankle Society (AOFAS) scores.

### Statistical analysis

The paired Student’s t-test was used to compare pre- and post-operative values. All data passed the normality and equal variance Shapiro–Wilk tests. *P* < 0.05 was considered statistically significant.

## Results (Tables 1 and 2)

### Imaging evaluations

The AP mortise angle during weight-bearing improved from a mean preoperative value of 80.5° to a mean postoperative value of 98.0°. The ML mortise angle during weight-bearing also improved from a mean preoperative value of 76.0° to a mean postoperative value of 85.0°. Additionally, the talar tilt angle on ankle stress radiography improved from a mean preoperative value of 19.5° to a mean postoperative value of 3.0°, while the anterior translation of the talus improved from a mean preoperative value of 27 mm to a mean postoperative value of 2.6 mm.

**Table 1:** Before and after surgery comparison

	Before surgery	After surgery	*p* value
Antero-posterior mortise angle during weight-bearing	80.5 ± 2.8°(77–86°)	98.0 ± 2.1° (96–102°)	<.0001
Lateral mortise angle during weight-bearing	76.0 ± 2.6° (74–82°)	85.0 ± 1.6° (83–88°)	<.0001
Stress radiography			
Talar tilt angle	19.5 ± 2.9° (14–24°)	3.0 ± 0.8° (2~4°)	<.0001
Anterior translation of the talus	27 ± 4.2 mm (16–32 mm)	2.6 ± 0.7 mm (2~4 mm)	<.0001
ROM			
Dorsiflexion	7.0 ± 4.3°(0–15°)	6.0 ± 4.0° (0–15°)	.109
Plantarflexion	45.0 ± 8.2° (25–60°)	40.0 ± 8.4° (20~60°)	.062
VAS	9.1 ± 1.4 (6–10)	1.6 ± 2.4 (0–6)	<.0001
AOFAS score	35.5 ± 8.6 (30–58)	88.4 ± 10.7 (58~100)	<.0001

**Table 2:** MRI signal changes before and after surgery

	Before surgery	After surgery
Signal change of MRI		
T1W	Observed	Disappeared, 9 patients (42.9%)
	21 patients	Reduced, 12 patients (57.1%)
T2W	Observed	Disappeared, 4 patients (19.1%)
	21 patients	Reduced, 17 patients (80.9%)

All patients showed improvements in MRI T1-weighted image signal changes, with a disappearance of preoperative signal changes and a reduction in the signal change area in 9 and 12 patients, respectively; additionally, in MRI T2-weighted images, there was a disappearance of preoperative signal changes and a reduction in the signal change area in 3 and 18 patients, respectively.

None of the patients demonstrated radiographic evidence of arthropathic changes in the peripheral joints.

### Clinical evaluation

The ROM was preserved, with similar pre- and post-operative values. The VAS improved from a preoperative mean of 9.1 points to a postoperative mean of 1.6 points. Additionally, the AOFAS improved from a preoperative mean of 35.5 points to a postoperative mean of 88.4 points.

There were 14 superficial pin-tract infections, which were treated with empirical oral antibiotics and daily pin-tract dressings. None of the patients experienced skin disorders that required additional surgery, deep infection, deep venous thrombosis, nerve palsy, adjacent joint disorders, or arthrofibrosis. One patient (79-year-old female) was using a crutch to walk by 5 years after surgery. One patient transiently experienced mild postoperative pain at the iliac crest; however, this had disappeared at the latest follow-up.

## Discussion

Various options exist for the surgical treatment of ankle osteoarthritis. Selection of the optimal treatment requires a thorough consideration of the patient’s characteristics. In patients with progressive or end-stage ankle osteoarthritis, total ankle arthroplasty is indicated for cases with bilateral involvement or degeneration in the adjacent joints. However, total ankle arthroplasty is contraindicated in patients with infectious ankle osteoarthritis or severe deformities (≥15° varus and valgus deformity of the ankle joint); it is also not appropriate for patients with a high level of physical activity (including sports and farming), even if they are ≥60 years of age [18]. Although ankle arthrodesis shows stable long-term outcomes and is effective in reducing pain, it has certain disadvantages including a loss in the ankle ROM and adjacent joint disorders. Furthermore, in countries such as Japan where people do not wear shoes and tend to sit on the floor in the house, patient satisfaction with ankle arthroplasty is relatively low [7, 9]. LTO includes valgus correction of the alignment and an outward shift of the weight-bearing line. The procedure has been reported to have good outcomes in patients with stage I-IIIA ankle osteoarthritis. However, LTO, which involves extra-articular osteotomy, is contraindicated in patients with ankle joint instability and may require additional surgery, such as ligament reconstruction [1, 19, 20].

Distraction arthroplasty includes cell mobilization from the bone marrow in the talus and tibial mortise (via a microfracture procedure or drilling), and requires treated patients to perform articulation while wearing an external fixator, allowing for an increased ROM. Joint traction for an appropriate period of time prevents damage to the regenerated tissue, and articulation promotes its maturation [18, 21]. In this study, we combined DTO and distraction arthroplasty. DTO has been shown to be effective in older patients with a high level of physical activity, as it preserves the ROM [22, 23]. In a study, DTO was successfully performed in patients with stage IIIB arthropathy and ankle joint instability. DTO offers certain advantages over arthrodesis, which include preservation of joint function and pain reduction. Another merit is that it exerts less influence on peripheral joints, which often cause problems in fixation. Hence, none of the patients in the present study had an adjacent joint disorder.

Deliberate flexion of the osteotomy serves to stabilize and provide more coverage to the talus. Since most patients with ankle osteoarthritis lack dorsiflexion, many surgeons are hesitant to flex the osteotomy and increase equinus. However, we overcame this issue by employing transverse Vulpius gastrocsoleus recession for increased equinus, which provided better coverage of the talus and shifted the healthier posterior cartilage anteriorly. This approach may have contributed to the observed good results.

The advantage of DTO over LTO is that it improves ankle joint stability by an angled osteotomy of the proximal tibial attachment site of the anterior tibiofibular ligament with valgus correction [23]. Without fibular osteotomy, DTO is similar to LTO with fibular osteotomy, in that they both correct alignments. Both osteotomies may shift the weight-bearing axis laterally by angulation of the osteotomized distal part of the tibia. However, only DTO without fibular osteotomy can narrow the lateral mortise in cases of medial ankle arthritis with mortise widening [22]. Therefore, DTO with joint distraction using a circular external fixator may also be beneficial to the cartilage [10, 24].

There are numerous reports on supramalleolar osteotomy with or without fibular osteotomy for varus ankle arthritis. Hongmou et al. [25] reported that fibular osteotomy may be necessary in supramalleolar osteotomy cases with a large talar tilt and small tibiocrural angles. Stufkens et al. [26] also reported that only supramalleolar osteotomy with fibular osteotomy shifts the pressure laterally for varus ankle arthritis. However, further research is required on this subject.

Since long-term non-weight-bearing leads to reduced walking ability in older patients, walking with a circular external fixator with strong fixation immediately after surgery may greatly benefit them and mechanical stimulation by weight-bearing may have additional effects. Conversely, DTO using a plate requires 1 to 2 months of non-weight-bearing [22, 23]. Caution is required with higher degrees of correction as it places a greater burden on soft tissues.

**Fig. 7:**
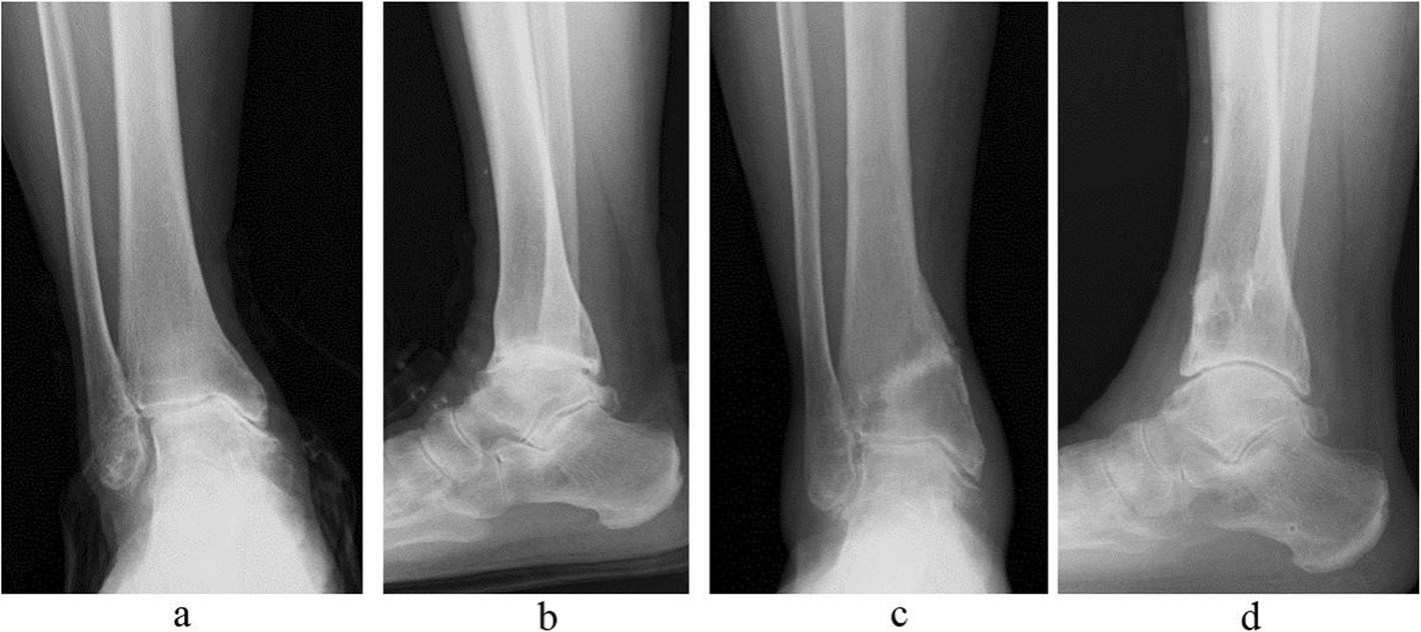
79-year-old female with ankle osteoarthritis (stage IIIB). **a** and **b** Before surgery. **c** and **d** After surgery. Narrowing of the joint space has been reduced

In our study, the evaluation of joint space narrowing on pre- and postoperative radiographs permitted the visualization of postoperative improvements with our technique (Fig. 7). Furthermore, MRI evaluations confirmed the improvements, with reductions or disappearance of preoperative signal changes after surgery.

This study has certain limitations. First, patients may find the use of a circular external fixator uncomfortable. However, one of the major reasons explaining the absence of deep infections or soft tissue complications requiring additional surgery in this cohort, may be the avoidance of plate fixation. Additionally, improvement of talus instability without ligament reconstruction requires a relatively large opening (i.e., about 20 mm) at the osteotomy site in most patients; this substantially increases the tension on the medial soft tissue in most patients. Therefore, additional studies with a larger number of older patients with ankle osteoarthritis and a high level of physical activity, are needed to validate the suitability of DTO with distraction arthroplasty using a circular external fixator as a treatment option for end-stage ankle osteoarthritis.

## Conclusions

In the treatment of patients with ankle osteoarthritis, it is important to consider the patient’s age and physical activity level while selecting the optimal surgical strategy.

DTO with joint distraction may be useful as joint-preserving surgery for medial ankle osteoarthritis in young patients, and in older patients with a high level of physical activity.

## Data Availability

The datasets used and/or analyzed during the current study are available from the corresponding author on reasonable request.

## Abbreviations

AOFAS: The American Orthopedic Foot & Ankle Society
AP: Antero-posterior
DTO: Distal tibial osteotomy
LTO: low tibial osteotomy
ML: Medio-lateral
MRI: Magnetic resonance imaging
PACS: The Patient Archiving and Communication System
ROM: Range of motion
VAS: Visual Analogue Scale
